# Development and Evaluation of a Case-Based Serious Game for Diagnosis and Treatment Planning in Orthodontic Education: Quasi-Experimental Study

**DOI:** 10.2196/73956

**Published:** 2025-08-27

**Authors:** Rochaya Chintavalakorn, Chayuth Chanwanichkulchai, Napat Buranasing, Napatsaporn Parivisutt, Naruchol Patchasri, Kawin Sipiyaruk

**Affiliations:** 1 Department of Orthodontics, Faculty of Dentistry, Mahidol University, 6 Yothi Road, Ratchathewi, Bangkok, 10400, Thailand, 66 22007813; 2 Doctor of Dental Surgery Program, Faculty of Dentistry, Mahidol University, Bangkok, Thailand

**Keywords:** dental education, gamification, orthodontics, serious game, simulation

## Abstract

**Background:**

Orthodontic education requires effective training in diagnosis and treatment planning, but traditional teaching methods may lack engagement and opportunities in offering a safe learning environment. Serious games are gaining momentum in dental education due to their positive educational impact in enhancing learner knowledge and motivation. However, their application in orthodontic diagnosis and treatment planning training remains unexplored.

**Objective:**

The aim of this study was to develop and evaluate a simulation-based serious game for training orthodontic diagnosis and treatment planning in virtual patients (OrthoVirt), examining its impact on student knowledge and satisfaction. This study also explored whether prior gaming experience influenced learning outcomes.

**Methods:**

A quasi-experimental study was conducted with 32 fourth-year dental students, who were requested to complete a preknowledge assessment, 3 simulated patients within OrthoVirt, a postknowledge assessment, and a satisfaction survey. Participants were categorized as gamers (n=16) or nongamers (n=16) based on self-reported weekly gaming time. The primary outcome was knowledge improvement, analyzed using 2-tailed paired *t* tests (Cohen *dz*). Group comparisons were conducted using 2-tailed independent *t* tests (Cohen *d*). User satisfaction was measured using a validated questionnaire based on the technology acceptance model. A stricter significance threshold (*P*<.01) and effect size metrics (Cohen *d* and Cohen *dz*) were used to account for the small sample size, multiple comparisons, and exploratory nature of the study.

**Results:**

Both gamer and nongamer groups showed significant knowledge improvement after using OrthoVirt (mean score increased from 10.75 (SD 2.75) to 14.75 (SD 1.81) out of 20; *P*<.001). The mean scores of the gamer group increased from 10.31 (SD 3.07) to 15.19 (SD 1.83) while those of the nongamer group rose from 11.19 (SD 2.40) to 14.31 (SD 1.74). No statistically significant differences were found between groups in pre- and postknowledge assessments as well as improvement scores (*P*>.01), suggesting that the educational benefit was consistent regardless of gaming background. Participants from both groups rated OrthoVirt positively, particularly for “perceived ease of use.” However, “perceived enjoyment” was rated slightly lower than other aspects, with nongamers scoring it 3.60 (SD 0.81) and gamers 3.45 (SD 0.73) out of 5, indicating a potential area for design enhancement. Overall satisfaction ratings were similar between the 2 groups (*P*>.01).

**Conclusions:**

OrthoVirt demonstrated potential as a supplementary tool for diagnosis and treatment planning in orthodontic education, with statistically significant improvements observed in learner knowledge. While feedback was generally positive, these findings should be interpreted with caution due to the quasi-experimental design and small sample size. Future development should focus on improving user enjoyment and engagement through entertaining design elements. Further research should explore how OrthoVirt can be integrated as a case discussion tool alongside lectures, with the potential to enhance learning not only in orthodontic education but also across other areas of dental training.

## Introduction

In orthodontic practice, diagnosis and treatment planning play a pivotal role in providing patient care and treatment outcomes. This competency is considered an essential component of the expected learning outcomes required for orthodontic practice [1]. Accuracy in diagnosis and treatment planning necessitates a thorough consideration of comprehensive patient information, encompassing various orthodontic needs, such as dental alignment, occlusion, skeletal relationships, and soft tissue considerations [2,3]. To accomplish this, orthodontists rely on the examination and analysis of diagnostic records, including radiographs, photographs, and dental impressions [4,5]. Not only current dental issues but also potential future growth and development are important to ensure optimal results and long-lasting stability. Therefore, effective delivery methods are necessary to equip dental professionals with this competence.

A variety of teaching and learning approaches are important in improving the complex skills required for diagnosis and treatment planning in orthodontics [1]. While traditional lectures provide basic knowledge, case-based learning offers invaluable opportunities for students to apply theoretical concepts to real-world scenarios, improving their critical thinking and decision-making abilities [6-8]. Integrating technology-enhanced learning tools, such as simulations and serious games, further enriches the educational experience, allowing students to explore immersive case scenarios and receive immediate feedback [9-12]. Using a combination of instructional methods can help students develop the full range of skills needed for effective orthodontic diagnosis and treatment planning. However, many current instructional methods tend to teach diagnostic skills separately, without providing a cohesive, interactive setting that mirrors the complexity of real-life orthodontic evaluation. This highlights the need for a simulation-based tool, designed specifically for orthodontic education, that can guide students through the step-by-step reasoning process involved in accurate diagnosis and treatment planning.

Serious games have currently gained momentum in dental education due to their positive educational impact across several aspects [13-16]. By immersing learners in realistic scenarios, serious games facilitate experiential learning, allowing students to apply theoretical knowledge to practical situations while refining clinical decision-making skills [17-19]. Furthermore, the gamified elements inherent in these educational tools can significantly enhance motivation and engagement among students, as they are incentivized to progress through challenges and receive immediate feedback [20,21]. In addition, serious games provide a safe learning environment where students can learn from their mistakes without real-world consequences [13,22,23]. As a result, serious games emerge as a promising interactive learning platform, providing dental learners with immersive experiences to practice orthodontic diagnosis and treatment planning. Despite these advantages, most serious games developed for dental education have focused on foundational sciences, communication training, or procedural skills. Few have been designed to support the clinical reasoning process in orthodontics, which requires multiple sources of diagnostic data, such as patient interviews, cephalometric analysis, and dental models, that are essential for accurate orthodontic diagnosis and treatment planning.

While simulation-based serious games have shown promise in dental education, few have addressed the unique complexity of orthodontic diagnosis and treatment planning, which demands the interpretation of multidimensional clinical records and synthesis of skeletal, dental, and soft tissue information [12,24]. Integrating serious games into this context offers undergraduate dental learners the opportunity to enhance critical thinking skills and apply theoretical knowledge in simulated clinical scenarios [25]. However, the effective adoption of serious games in education may not be uniform across all learners [20,26]. As serious games often incorporate interactive features and mechanics similar to those found in video games, prior gaming experience may influence how students engage with and benefit from these educational tools [27-29]. Learners with higher levels of gaming familiarity may find serious games more engaging, whereas those with less experience might encounter greater cognitive load or usability challenges, potentially impacting their learning outcomes.

Given these considerations, it is important to determine whether the educational benefits of serious games are equally practical to learners with diverse gaming experiences in dental education. Consequently, to serve as a supplementary tool rather than a replacement for conventional orthodontic education, this research aimed to design and evaluate a serious game for training diagnosis and treatment planning in orthodontic practice, comparing its educational impact between gaming and nongaming users. The evidence retrieved from this research may provide valuable insights into the potential benefits and practical considerations of integrating serious games, paving the way for future innovations in teaching and learning in orthodontic education.

## Methods

### Game Development

#### Game Concept

A simulation-based serious game for training orthodontic diagnosis and treatment planning in virtual patients (OrthoVirt) was designed and developed using “React,” an open-source JavaScript framework. Within the game, learners were allowed to interact with simulated patients with orthodontic concerns. The development of OrthoVirt was driven by Kolb’s [30] Experiential Learning Theory, which frames learning as a continuous cycle of doing, reflecting, thinking, and applying. The structure of the game reflects the clinical process in orthodontic practice, from gathering patient information to formulating a diagnosis and treatment plan. By working through these stages, learners can gain experience through active learning in a simulated environment. As they receive feedback and revisit their decisions, learners are encouraged to reflect on their thought processes and refine their clinical judgment, helping them improve competencies in diagnosis and treatment planning.

#### Instructional Design and Learning Tasks

The game login requires a user account generated from a secure back-office system by administrators. Following a log-in process, learners could opt to proceed with the game with 1 of the 3 simulated patients with different orthodontic problems, including deep bite, crossbite, and open bite (learning scenarios). Each scenario began with the patient’s chief complaint and involved 5 tasks, which were Task 1: Patient interview, Task 2: Intraoral and extraoral examination, Task 3: Model analysis, Task 4: Radiographic examination, and Task 5: Diagnosis and treatment planning.

The game tasks were designed using a structured and interactive instructional approach to enhance learner competency in orthodontic diagnosis and treatment planning through realistic patient-case scenarios. Learners begin with Task 1, which involves interviewing a simulated patient to understand the chief complaint and gather general information. Completion of this task unlocks Task 2, where students conduct intraoral and extraoral examinations based on standardized visuals of the patient. Once these examinations are completed, students gain access to Tasks 3 and 4, which can be approached in either order. Task 3 presents high-resolution images of dental models, enabling learners to evaluate occlusal relationships, overjet, overbite, and arch form. Task 4 provides cephalometric radiographs accompanied by premeasured angular and linear values. Importantly, while these measurements are provided, learners are expected to interpret them independently, promoting critical thinking and reinforcing the kind of clinical judgment expected in orthodontic practice. As learners progress through each diagnostic step, a dynamic information box compiles key findings, allowing for easy review and synthesis. In the final stage, Task 5, learners draw on all prior assessments to formulate a comprehensive orthodontic diagnosis and propose an appropriate treatment plan. This sequential process mirrors the structure of authentic clinical workflows and encourages deep engagement with each component of the diagnostic pathway.

#### Game Interface

**Figure 1.**
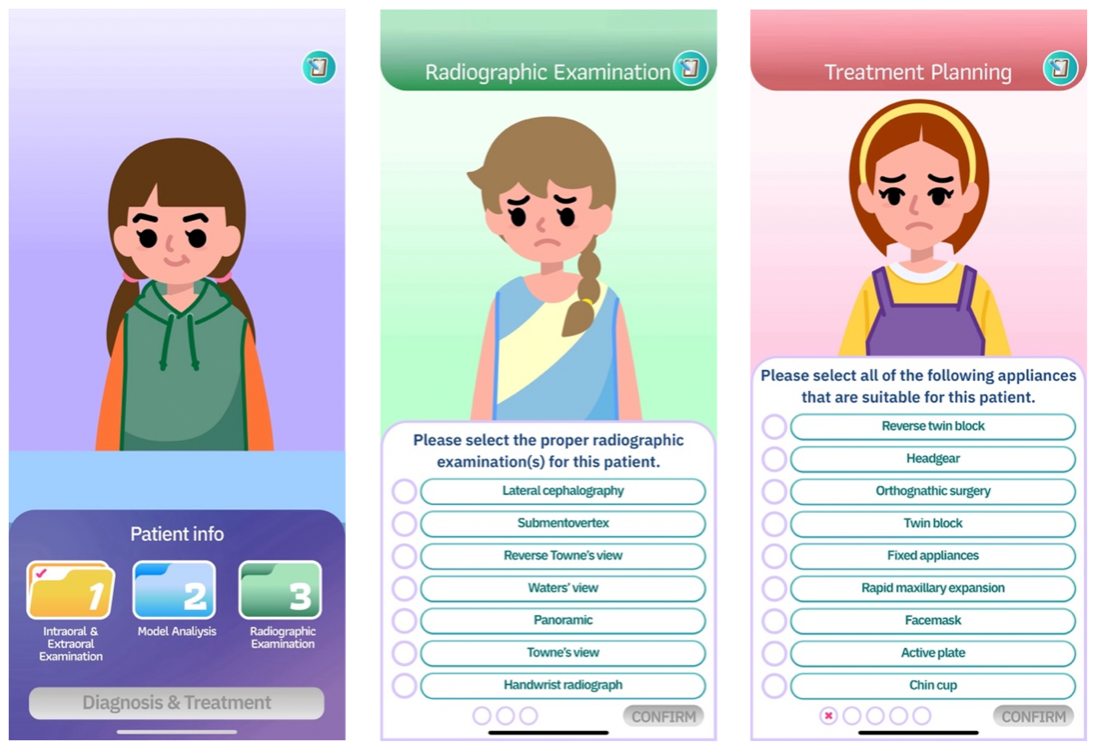
Visual representations of the game interface within OrthoVirt.

As presented in Figure 1, the game interface guides students through a structured diagnostic workflow that mirrors clinical practice. The first screen introduces the simulated patient and provides access to key diagnostic stages aligned with the game’s intended learning outcomes. In the radiographic examination task, learners apply their knowledge of diagnostic imaging to select appropriate radiographic views, reinforcing concepts taught in preclinical orthodontic courses. The final screen presents a treatment planning scenario, where students must integrate diagnostic findings and theoretical understanding to select suitable appliances. These interactive steps are designed to promote clinical reasoning and strengthen foundational knowledge in orthodontic diagnosis and treatment planning.

#### Feedback System

**Figure 2.**
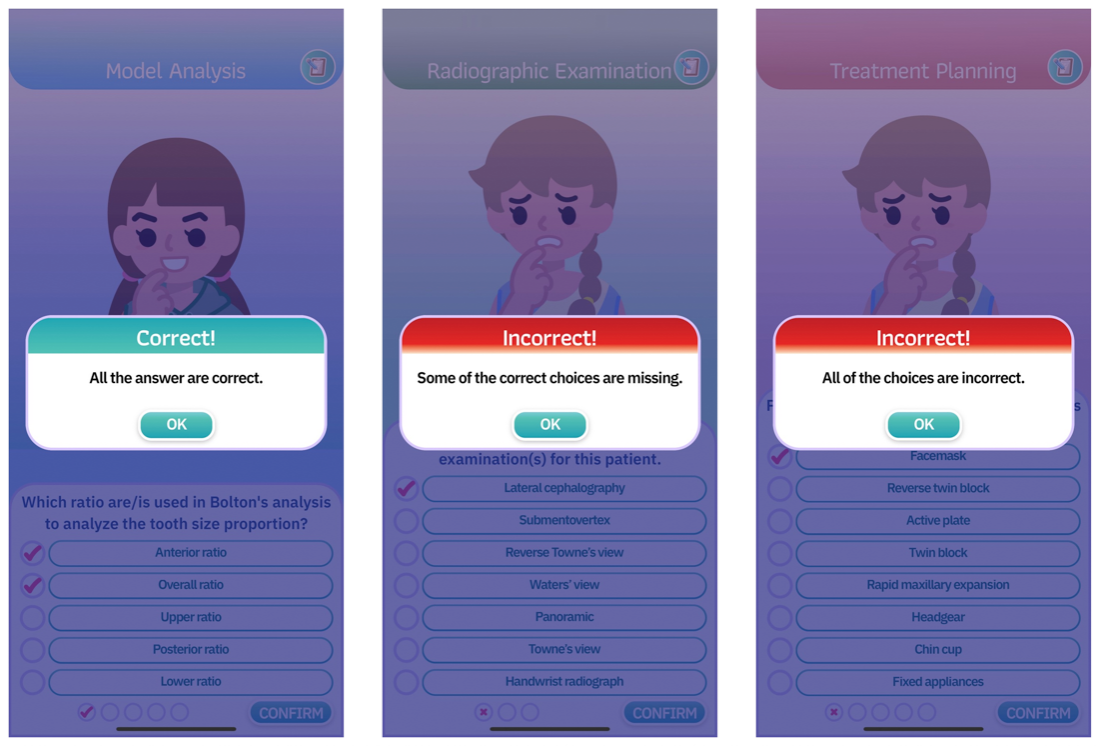
Examples of feedback formats provided within the game.

The game incorporated a feedback mechanism that provided learners with immediate responses in diverse formats following their interactions. Notably, the game offered informative feedback explaining the clinical rationale behind each question, regardless of whether the response was correct. In addition, clues were provided to learners in instances where incorrect responses were selected. Furthermore, the game deducted scores and reflected them through the facial expressions of the simulated patients when learners chose incorrect answers. As shown in Figure 2, the feedback system differentiated between levels of error. For example, it indicated whether only some answers were incorrect or if all choices were wrong, thereby tailoring the guidance to the nature of the mistake. These feedback elements were designed to support learner reflection and maintain engagement throughout the learning process.

### Research Design

This study used a quasi-experimental research design to assess the impact of OrthoVirt in orthodontic education, comparing gaming and nongaming users. The participants voluntarily involved fourth-year dental undergraduates from the Faculty of Dentistry, Mahidol University. Participants initially were assigned to complete a preknowledge assessment. Following a 7-day interval, they engaged with OrthoVirt, completing 3 orthodontic scenarios within the game. Immediately after, participants were required to complete a postknowledge assessment to measure knowledge acquisitions. In addition, participants filled out a survey regarding their experience with OrthoVirt. To maintain data integrity, students were instructed to work independently on all tasks without peer discussion.

### Participants and Recruitment

The research participants included fourth-year dental undergraduates at the Faculty of Dentistry, Mahidol University. However, participants who had not passed the course “Diagnosis and Treatment Planning in Orthodontics" during the 2023 academic year were excluded from the research. This was to ensure a consistent baseline of relevant orthodontic knowledge and because the game was designed as a supplementary tool to reinforce content from that course. The participants were categorized into 2 groups: gaming users, who played video games for at least 1 hour per day (7 h/wk), and nongaming users, who played for less than this duration. Participants were recruited for this research using voluntary sampling. This nonprobability sampling technique was considered appropriate for the study, despite potential concerns about nonresponse bias [31]. Participants voluntarily engaged in the learning activity, which led to accurate and reliable research findings with a low possibility of dropout [32].

To determine the appropriate number of participants, a statistical calculation was performed using a method suitable for comparing paired datasets, aligning with the primary objective of assessing knowledge acquisition following OrthoVirt use [33]. The calculation assumed a large effect size (Cohen *dz*=1.425), based on a previous study [26]. The calculation indicated that to detect a significant difference in scores between the 2 knowledge assessments, with 95% power and a 1% significance threshold, each group required at least 13 participants. Anticipating potential participant attrition, this number was adjusted upward by 20%, resulting in a target of 16 participants per group. Consequently, this study recruited 32 dental students, comprising 16 gaming users and 16 nongaming users. A 1% significance level (*P*<.01) was adopted to reduce the risk of Type I error, considering the relatively small sample size, multiple comparisons, and the exploratory nature of the study.

### Outcome Measurements

#### Knowledge Assessments

Participants were required to complete pre- and postknowledge assessments to assess knowledge improvement. The initial development of the knowledge assessment was performed by a research team member with expertise in orthodontics (RC). The assessment was reviewed by 3 experts in orthodontics who were not involved in the study to ensure the content validity and its alignment with the expected learning outcomes of the game. Each assessment included 20 multiple-choice questions. The content reflected a wide range of cognitive skills relevant to orthodontic diagnosis and treatment planning. These included dental and skeletal classification, growth assessment, cephalometric interpretation, space analysis, and treatment selection. Questions were designed to test not only factual knowledge but also clinical reasoning. Specifically, the assessment items spanned multiple levels of Bloom’s cognitive domain, ranging from “Remembering and Understanding” to “Applying and Analyzing.” Several questions required learners to interpret diagnostic images or synthesize measurements to draw clinical conclusions, thereby promoting higher-order thinking aligned with real-world orthodontic decision-making. Examples of assessment items and their corresponding Bloom’s cognitive domains are provided in Multimedia Appendix 1. To reduce potential memory effects, both question order and answer choices were randomized between the 2 assessments.

#### User Satisfaction

The paper-based questionnaire was constructed to gather user satisfaction based on the technology-acceptance model [34], with questions adapted from existing literature on serious games [25,35,36]. Three experts in health care education were requested to validate the questionnaire using content validity, with items iteratively refined until each achieved an item-objective congruence index above 0.5. Based on the internal consistency reliability assessment, items with low performance were removed until all constructs achieved Cronbach α coefficients above 0.7 (Perceived usefulness: 0.714, Perceived ease of use: 0.727, and Perceived enjoyment: 0.803). The validated questionnaire consisted of 18 five-point Likert scales (Multimedia Appendix 2), covering “perceived usefulness (6 items),” “perceived ease of use (6 items)” and “perceived enjoyment (6 items),” with responses ranging from “Strongly disagree” (1) to “Strongly agree” (5).

### Data Analysis

The research data were analyzed using the IBM SPSS Statistics (SPSS version 29, IBM Corp). The distribution of the data was approximately normal, and the assumption of equal variance for the independent *t* test was met, supporting the use of parametric statistical analyses. Descriptive statistics were used to summarize the data. Cognitive improvement was assessed using a paired *t* test, while a comparison of knowledge acquisition between the gaming and nongaming groups was made using an independent *t* test. The threshold for statistical significance was set at *P*<.01. In addition, effect sizes were reported using Cohen *d* for independent *t* tests and Cohen *dz* for paired *t* tests to quantify the magnitude of observed effects.

### Ethical Considerations

This research protocol was reviewed and approved by the Institutional Review Board of Faculty of Dentistry/Faculty of Pharmacy, Mahidol University (the certificate of approval number: MU-DT/PY-IRB 2024/012.0103). All methods were performed in accordance with the relevant guidelines and regulations. Informed consent was obtained from all participants prior to their involvement in the study. They were clearly informed about the research purpose, procedures, and their rights, including the right to withdraw at any time. To protect participants’ privacy and confidentiality, all collected data were anonymized prior to analysis. Participants did not receive financial compensation or material incentives for their participation. However, they were provided with an opportunity to learn from the OrthoVirt game, which was positioned as an educational activity to support their academic development. No identifiable images of participants are included in the paper or supplementary materials.

## Results

### Research Participants

A total of 32 dental students participated in the study, comprising 26 females and 6 males. The participants reported a mean gaming time of 9.59 (SD 13.61) hours per week, with a range of 0‐63 hours. Among the participants, 16 were classified as gaming users, playing an average of 17.63 (SD 15.56; range 7‐63) hours per week. The remaining 16 were categorized as nongaming users, with a mean gaming time of 1.56 (SD 1.71; range: 0‐5) hours per week. The gamer group included 4 male and 12 female participants, while the nongamer group included 2 male and 14 female participants. All participants were enrolled in the same academic year (fourth year) and were therefore presumed to be of similar age.

### Knowledge Acquisition

According to paired *t* test analysis, both gamer and nongamer groups exhibited statistically significant improvements in their assessment scores (*P*<.001; Cohen *dz*=1.65), as shown in Table 1. The gamer group demonstrated an increase in mean score from 10.31 (SD 3.07) to 15.19 (SD 1.83), with a large effect size (Cohen *dz*=1.82). Similarly, the nongamer group showed improvement, with mean scores rising from 11.19 (SD 2.40) to 14.31 (SD 1.74), yielding a large effect size (Cohen *dz*=1.45). These results indicate substantial performance enhancement in both groups following the intervention.

**Table 1.** Analysis of pre- and postknowledge assessment scores.

Groups	Preknowledge assessment^a^ Mean (SD)	Postknowledge assessment^a^ Mean (SD)	Mean difference (95% CI)	*P* value^b^
Gamer group	10.31 (3.07)	15.19 (1.83)	4.88 (3.23-6.52)	<.001
Nongamer group	11.19 (2.40)	14.31 (1.74)	3.12 (1.53-4.72)	<.001
Total	10.75 (2.75)	14.75 (1.81)	4.00 (2.87-5.12)	<.001

a The maximum possible score for both knowledge assessments was 20.

b All *P* values are 2-tailed, with significance set at *P*<.01.

### Comparison of Knowledge Acquisitions Between Groups

An independent *t* test was used to compare knowledge acquisition between the gamer and nongamer groups (Table 2). No statistically significant differences were observed in preknowledge assessment, postknowledge assessment, or score improvement between the 2 groups (*P*>.01 for all comparisons). The corresponding effect sizes were small to moderate, that is, Cohen *d*=0.32 for preknowledge assessment, Cohen *d*=0.49 for postknowledge assessment, and Cohen *d*=0.57 for score improvement. These results suggest that gaming experience did not have a significant impact on knowledge gains from the intervention.

**Table 2.** Comparison of knowledge acquisitions between gamer and nongamer groups.

Assessments^a^	Gamer group Mean (SD)	Nongamer group Mean (SD)	Mean difference (95% CI)	*P* value^b^
Preknowledge assessment	10.31 (3.07)	11.19 (2.40)	0.88 (–2.87 to 1.12)	.38
Postknowledge assessment	15.19 (1.83)	14.31(1.74)	–0.88 (–0.42 to 2.17)	.18
Score improvement	4.88 (3.10)	3.13 (2.99)	–1.75 (–0.45 to 3.95)	.11

a The maximum possible score for both knowledge assessments was 20.

b All *P* values are 2-tailed, with significance set at *P*<.01.

### User Satisfaction

Both gamer and nongamer groups rated OrthoVirt favorably across all assessed aspects (Table 3). An independent *t* test showed no statistically significant differences in satisfaction scores between the 2 groups across all aspects (*P*>.01 for all comparisons). The highest-rated aspect in both groups was “perceived ease of use,” with mean scores of 4.07 (SD 0.54) and 4.06 (SD 0.61) out of 5 for nongamer and gamer groups, respectively (Cohen *d*=0.02). Perceived usefulness was also rated highly, with scores of 3.93 (SD 0.42) for nongamers and 3.98 (SD 0.54) for gamers (Cohen *d*=0.10). In contrast, “perceived enjoyment” received slightly lower ratings compared to other aspects, with nongamers scoring it 3.60 (SD 0.81) and gamers 3.45 (SD 0.73; Cohen *d*=0.10).

**Table 3.** Comparison of satisfaction scores between gamer and non-gamer groups.

Aspects^a^	Gamer group Mean (SD)	Nongamer group Mean (SD)	Mean difference (95% CI)	*P* value^b^
Perceived usefulness	3.98 (0.54)	3.93 (0.42)	–0.05 (–0.30 to 0.40)	.77
Perceived ease of use	4.06 (0.61)	4.07 (0.54)	0.01 (–0.42 to 0.41)	.98
Perceived enjoyment	3.45 (0.73)	3.60 (0.81)	0.15 (–0.71 to 0.41)	.59
Overall	3.83 (0.55)	3.86 (0.52)	0.03 (–0.42 to 0.35)	.85

a User satisfaction was measured on a five-point Likert scale, ranging from “Strongly disagree” (1) to “Strongly agree” (5).

b All *P* values are 2-tailed, with significance set at *P*<.01.

## Discussion

### Knowledge Gains and Educational Value

The implementation of simulation-based serious games in orthodontic education has shown promising results in cognitive improvement. This research demonstrated significant knowledge improvements after engaging with OrthoVirt, aligning with previous research highlighting the efficacy of serious games as educational tools [20,22,25,37-40]. The observed knowledge improvement, evidenced by increased assessment scores, can be attributed to the game’s dynamic learning environment, which allows students to learn from mistakes without real-world consequences [14,35,41]. While other games have demonstrated knowledge improvements in other areas, OrthoVirt is designed specifically for orthodontic education, incorporating discipline-specific data, such as model analysis and cephalometric interpretation. In contrast to studies comparing serious games with traditional instruction, this study adopted a quasi-experimental design comparing gamer and nongamer groups to assess the game’s applicability across varied learner profiles. Satisfaction results further support the educational value of the game. These results suggest that OrthoVirt represents a valuable addition to traditional orthodontic education methods, offering an engaging and effective platform for knowledge acquisition in orthodontic diagnosis and treatment planning. In addition, its core design may be adaptable for use in other areas of dental education that emphasize diagnostic reasoning and clinical decision-making.

The absence of significant differences in knowledge gain between gamer and nongamer groups suggests that the educational benefits of OrthoVirt are accessible to a diverse student population, regardless of prior gaming experience. This finding is particularly important as it indicates the broad applicability of the tool, consistent with existing evidence [42,43]. However, it is worth considering that gamers might experience lower stress levels compared to nongamers, potentially due to their ability to relieve stress through gaming and their higher tolerance for electronic devices [44,45]. Furthermore, the intrinsic motivation generated by games may inspire students in the gamer group to engage more deeply with the learning process, which could explain why this group tends to show greater improvement after using OrthoVirt [46]. However, these factors were not directly measured in this study; thus, while such explanations are theoretically plausible, they remain speculative. These observations underscore the potential of serious games to enhance knowledge and positively influence the learning experience across diverse student profiles in dental education.

### User Experience and Satisfaction

The user experience and satisfaction with OrthoVirt were evaluated, with a particular focus on its ease of use, yielding insights into the game’s effectiveness and areas for potential improvement. The satisfaction level regarding ease of use was rated highest by both gamer and nongamer groups, suggesting that users found the interface intuitive and user-friendly, which is crucial for educational software adoption, including serious games [47,48]. This ease of use is a critical factor in serious games, as it reduces cognitive load associated with learning the software itself, allowing users to focus more on the educational content [49]. This reflects the alignments between the interactive functions and pedagogical design dimensions of the evaluation framework of serious games [20]. The volume of information presented in each section could potentially impact the perceived ease of use. This feedback highlights the delicate balance required in designing educational games that are both comprehensive and user-friendly [50,51]. The high ease of use score suggests that OrthoVirt successfully achieved this balance for most users despite these concerns. These findings underscore the importance of user-centered design in educational games, where ease of use plays a vital role in optimizing engagement and, consequently, educational outcomes.

The game functions in OrthoVirt, including interactive activities and feedback mechanisms, aimed at enhancing learner enjoyment and engagement. Interactivity facilitates easier gameplay and provides improved performance feedback, thereby augmenting the overall learning experience for the user [20,25,52]. However, the enjoyment satisfaction ratings were rated notably lower than the ease of use and usefulness. This finding suggests a gap in emotional and motivational engagement, potentially because learners may have compared this serious game to commercial entertainment games, which are primarily designed to maximize immersion and enjoyment. Considering the core difference from entertainment games, serious games are not solely focused on providing enjoyment but are designed to facilitate knowledge acquisition alongside engagement [53,54]. As a result, they may not foster the same level of intrinsic interest in their subject matter [51]. According to the review of Maxim and Arnedo-Moreno’s synthesis [55], effective serious game design involves the balance of educational content with motivational elements throughout 4 phases of exploration, design, development, and assessment. Therefore, these findings indicate a need for more integration of motivational and emotional engagement components during the design phase, even as usability and learning objectives were effectively addressed.

With the relatively lower enjoyment scores of OrthoVirt, the prioritization of enhancements that directly target motivational engagement should be recommended. As proposed in the exploration and design phases of the framework [55], understanding learner profiles and collaborative dynamics can support the integration of multiplayer elements or peer consultation tools, promoting social learning and critical thinking. First, incorporating social interaction features, such as competitive features or peer-to-peer interactivity, may promote deeper engagement and potentially reduce learner stress [56,57]. Future iterations could also explore collaborative consultation mechanisms, fostering critical thinking through group problem-solving and brainstorming, which leverages social constructivism principles [58,59]. These enhancements will address the identified enjoyment gap by fostering social and emotional connections. Adaptive features supported by artificial intelligence could personalize the difficulty level and learning path based on user performance, directly addressing user feedback related to engagement and enjoyment [60]. These enhancements could transform OrthoVirt into a more dynamic and engaging learning environment, in which further research could evaluate the impact of these proposed changes on learning engagement in dental education.

### Research Limitations

This study had limitations that should be considered. First, voluntary sampling may have introduced selection bias, as students more interested in technology-enhanced learning were more likely to participate. While this approach facilitated recruitment and ensured engagement, as evidenced by zero dropout, it may limit the generalizability of the findings. In addition, the gender imbalance in the sample (predominantly female) may further affect generalizability. Moreover, categorizing participants as gamers or nongamers based on self-reported gaming habits may have led to recall bias and misclassification. In addition, although using identical questions in the pre- and postknowledge assessments ensured consistent difficulty, some memory effect may still have occurred, despite efforts to mitigate this through shuffling item order. Furthermore, the absence of a control group limits direct comparisons between OrthoVirt and traditional methods. Academic performance data were not collected, which represents an additional limitation of this study. However, since OrthoVirt is designed as a supplementary educational tool rather than a standalone replacement, this limitation does not invalidate the exploratory value of the study. Importantly, as this research used a quasi-experimental design with voluntary participation and a relatively small sample, the ability to draw causal inferences or broadly generalize the findings is limited.

### Recommendations for Future Research

Future research should investigate how OrthoVirt can be integrated as a case discussion tool alongside traditional lectures, potentially enhancing the learning experience in orthodontic education. Further studies with larger and more diverse cohorts are required to confirm the findings of this study. In addition, research on the long-term retention of knowledge, as well as the impact of enhanced entertainment and social interaction elements, may inform improvements in learner engagement and broader applicability. Expanding the concept and underlying framework of OrthoVirt to other areas within dental education could also be considered.

### Conclusions

This study provides evidence that OrthoVirt can be used as a supplementary tool to support knowledge acquisition in orthodontic diagnosis and treatment planning. Statistically significant improvements in knowledge were observed following use of the game, with similar benefits reported regardless of prior gaming experience. While participants generally provided positive feedback on OrthoVirt, particularly highlighting its usefulness and ease of use, the study revealed opportunities for enhancement, especially in terms of user enjoyment. Future development of OrthoVirt should therefore prioritize improvements in entertainment and social interaction elements to further enhance learner engagement and motivation. These findings may also inform the development and integration of serious games across broader areas of dental education beyond orthodontics.

## Supplementary material

10.2196/73956 Multimedia Appendix 1 Examples of assessment items and their corresponding Bloom’s cognitive domains.

10.2196/73956 Multimedia Appendix 2 Satisfaction questionnaire toward the use of OrthoVirt.
