# Supplementary material for: Development and Evaluation of a Case-Based Serious Game for Diagnosis and Treatment Planning in Orthodontic Education: Quasi-Experimental Study
Source: JMIR Serious Games. 2025 Aug 27;13:e73956. doi: 10.2196/73956 (PMC12384673; doi:10.2196/73956)
Supplement: Multimedia Appendix 1 [file games-v13-e73956-s001.pdf]

Multimedia Appendix 1

Examples of assessment items and their corresponding Bloom's cognitive domains

Assessment items	Cognitive level
Which of the following analyses is used to determine intermaxillary tooth size proportion?<br>A. Tanaka and Johnston equation<br>B. Moyer's proportional table method<br>C. Bolton's analysis<br>D. Nance analysis	Remembering
Please select the correct statement regarding Angle's classification of malocclusion.<br>A. If the distobuccal cusp of the upper first molar occludes with the mesiobuccal groove of the lower first molar, it is classified as Angle's Class I.<br>B. If the distobuccal cusp of the upper first molar occludes distal to the mesiobuccal groove of the lower first molar, it is classified as Angle's Class II.<br>C. If the mesiobuccal cusp of the upper first molar occludes mesial to the mesiobuccal groove of the lower first molar, it is classified as Angle's Class II.<br>D. If the mesiobuccal cusp of the upper first molar occludes mesial to the mesiobuccal groove of the lower first molar, it is classified as Angle's Class III.	Remembering

Assessment items	Cognitive level
In the case of a patient missing a lower incisor, which space analysis method is most appropriate?<br>A. Tanaka and Johnston equation<br>B. Moyer's proportional table method<br>C. Radiographic method<br>D. Lundström method	Understanding
What is the diagnosis for a patient with a retrognathic maxilla and prognathic mandible?<br>A. Skeletal Class I<br>B. Skeletal Class II<br>C. Skeletal Class III<br>D. Deep configuration	Understanding
Which appliance is typically indicated for managing posterior dental crossbite?<br>A. Twin block<br>B. Anterior bite plane<br>C. Reverse headgear<br>D. Lateral expansion plate	Applying
What is the appropriate treatment for an 11-year-old patient presenting with retroclined upper incisors and anterior crossbite?<br>A. Active plate with spring<br>B. Rapid palatal expansion<br>C. Twin Block<br>D. Headgear	Applying

Assessment items	Cognitive level
Based on the cephalometric values provided, how would you classify the maxilla?<br>*(Note: A cephalometric measurement table was shown to participants during the assessment but is not included here due to privacy restrictions.)*<br>A. Orthognathic maxilla<br>B. Retrognathic maxilla<br>C. Prognathic maxilla<br>D. Vertical maxillary excess	Analyzing
Please determine the facial profile of each provided patient from left to right.<br>*(Note: Figures were shown to participants during the assessment but are not included here due to privacy restrictions.)*<br>A. Concave, Straight, Convex<br>B. Convex, Straight, Concave<br>C. Straight, Concave, Convex<br>D. Concave, Convex, Straight	Analyzing
Based on the cephalometric data provided, which of the following statements is/are correct?<br>*(Note: A cephalometric measurement table was shown to participants during the assessment but is not included here due to privacy restrictions.)*<br>A. The skeletal type is Class II based on AO-BO measurements.<br>B. The patient exhibits mandibular prognathism based on B point–N perpendicular.<br>C. The mandible and maxilla are shorter than average based on Co-A and Co-Gn values.<br>D. The maxilla and mandible are proportionate based on the mandibular–maxillary difference.	Evaluating

Assessment items	Cognitive level
Which of the following is an inappropriate treatment plan for an 8-year-old patient with Class II Division I malocclusion?<br>A. Fixed appliances<br>B. Twin block<br>C. Facemask<br>D. Headgear	Evaluating
