# Supplementary material for: Development and Evaluation of a Case-Based Serious Game for Diagnosis and Treatment Planning in Orthodontic Education: Quasi-Experimental Study
Source: JMIR Serious Games. 2025 Aug 27;13:e73956. doi: 10.2196/73956 (PMC12384673; doi:10.2196/73956)
Supplement: Multimedia Appendix 2 [file games-v13-e73956-s002.pdf]

Multimedia Appendix B

Satisfaction questionnaire toward the use of OrthoVirt

Part 1: Perceived usefulness of OrthoVirt

Please rate how much you agree with each of the following statements regarding the usefulness of OrthoVirt.

Perceptions	1 Strongly disagree	2 Disagree	3 Neither agree or disagree	4 Agree	5 Strongly agree
The game helped me conduct a more comprehensive patient interview.	☐	☐	☐	☐	☐
The game helped me make more accurate orthodontic diagnoses.	☐	☐	☐	☐	☐
The game helped me develop appropriate orthodontic treatment plans.	☐	☐	☐	☐	☐
The content of the game thoroughly covered the course material.	☐	☐	☐	☐	☐
The game enhanced my overall knowledge in orthodontics.	☐	☐	☐	☐	☐
The game helped me identify areas where I need to improve in orthodontics.	☐	☐	☐	☐	☐

Part 2: Perceived ease of use of OrthoVirt

Please rate how much you agree with each of the following statements regarding the ease of use of OrthoVirt.

Perceptions	1 Strongly disagree	2 Disagree	3 Neither agree or disagree	4 Agree	5 Strongly agree
It was easy and simple to access the game.	☐	☐	☐	☐	☐
The game interface was easy to understand.	☐	☐	☐	☐	☐
The language used in the game was clear and easy to understand.	☐	☐	☐	☐	☐
The game provided clear instructions on how to complete each task.	☐	☐	☐	☐	☐
The gameplay mechanics were easy to follow.	☐	☐	☐	☐	☐
The navigation within the game was straightforward.	☐	☐	☐	☐	☐

Part 3: Perceived enjoyment of OrthoVirt

Please rate how much you agree with each of the following statements regarding the enjoyment of OrthoVirt.

Perceptions	1 Strongly disagree	2 Disagree	3 Neither agree or disagree	4 Agree	5 Strongly agree
I enjoyed playing the game.	☐	☐	☐	☐	☐
The game made me more enthusiastic about learning.	☐	☐	☐	☐	☐
The game kept my attention throughout.	☐	☐	☐	☐	☐
The changing facial expressions of the characters motivated me to continue playing.	☐	☐	☐	☐	☐
The game's visuals and colors made it more engaging.	☐	☐	☐	☐	☐
The game made learning feel less stressful.	☐	☐	☐	☐	☐
